# Research on Subsidy Strategy of Shared Accommodation Platform under the Background of Big Data Based on Evolutionary Game

**DOI:** 10.1155/2022/9066215

**Published:** 2022-04-01

**Authors:** Meng Xiao, Chang Zhai

**Affiliations:** School of Management, Shenyang University of Technology, Shenyang 110021, China

## Abstract

To attract customers and landlords to join the platform is an important problem to be solved when expanding the size of the shared accommodation platform, and subsidy policies have been proven to be an effective approach in many areas of sharing. In order to analyze the strategies of all parties in the subsidy policy of the shared accommodation platform, a three-party evolutionary game model of shared accommodation platform, consumers, and landlords was built. The strategy stability of each game subject was analyzed, and the equilibrium point stability was explored based on Lyapunov's method. The game model and influencing factors were simulated and analyzed by MATLAB 2016. Results demonstrated that the consumers and landlords were promoted to join the shared accommodation platform by increasing subsidies to consumers and landlords and reducing consumers' time costs and landlords' service fees from the early stage to the rapid development stage of the shared accommodation platform; with the increase of the proportion of landlord's share in expenses and the decrease of the opportunity loss of platform nonsubsidizing, the strategy of the platform gradually evolves from subsidy to nonsubsidy. The conclusions of this study provide guidance for the mature path of the shared accommodation platform and also give suggestions for the development of the sharing economy.

## 1. Introduction

The comprehensive application of emerging technologies such as the Internet and big data in the market brings special operation mode, effective realization tools, and high-quality value creation, which have opened up a new road for the development of market industries and accelerated the evolution of the global economic format. In recent years, benefiting from technological advances and the increasing awareness of people's awareness towards sustainability, the ranges of shared products have increased dramatically [1]. Sharing has turned from a “nonreciprocal prosocial behavior” [2] to a unique economic model [3], which brings innovation and efficiency to local development [4]. According to Juniper Research, the global market for the sharing economy is expected to reach $40.2 billion in 2022 [5]. Sharing platforms are springing up all over the world, and the emerging business model of “Internet Plus” is gradually becoming the important engine for global economic upgrading and transformation. The sharing economy has started to develop in many industries around the world, such as Airbnb, an online travel and housing rental company established in 2008, and Uber, an online car service established in 2009. As an important trend for future development [6], the sharing economy has had a profound impact on the social economy to change people's consumption behavior and consumption patterns [7] and develop many new models by integrating with various industries [8]. Among the industries, the development of the shared accommodation industry has been remarkable [9].

The traditional accommodation industry is an important sector of the service industry, providing entertainment and dining services to travelers. However, with rapidly changing market conditions and competition, shared accommodation is a new way for the industry to develop as a result of the deep integration of the accommodation industry with information technology. According to the “China Sharing Economy Development Report 2020,” the market size of shared accommodation in China was about 22.5 billion yuan, increasing of 36.4% over the previous year, and the revenue of the shared accommodation industry accounted for 7.3% of the total revenue of the accommodation industry in 2019. The growing shared accommodation industry is bound to have a profound impact on the development of the industry. Therefore, it is important to analyze the development law of shared accommodation and understand the model in the development stage, which will be guiding significance for promoting the shared accommodation industry and socioeconomic development.

As a disruptive innovation, shared accommodation has become an important model to allocate urban accommodation resources, revitalize the rural tourism industry, and activate the development potential of the service industry [10–12]. Some companies have already achieved success in shared accommodation, represented by HomeAway and Airbnb Inc. [13]. The research on shared accommodation has gradually increased since 2013, with the market size of shared accommodation expanding [14]. The development of the shared accommodation industry in many areas imitated Airbnb, but the process is difficult for the trust barriers and legal regulation problems among various stakeholders [15]. Some scholars have proposed that the development of shared accommodation in China draws on the successful experience of Airbnb [16, 17], but it also faces the dilemma of “acclimatization” [18]. Therefore, how to keep the market alive of shared accommodation has become a problem that needs to be solved by shared accommodation companies in the global.

Shared accommodation is a type of nonstandard accommodation in which homeowners rent out their idle rooms to travelers for a short period through an online network platform [19]. Compared to traditional accommodation, shared accommodation has a wider variety of room types and caters to travelers' desire to “live like a local” with an authentic destination experience [20], which makes it popular among customers [21]. There are three parties involved in shared accommodation: the landlord (the operator providing the room for rent), the shared accommodation platform, and the consumer (the renter) [22]. The shared accommodation platform is the intermediary connecting landlords and consumers, increasing the efficiency for matching and reducing the search costs. As the intermediary organization, the platform charges the service fee for both landlords and consumers. Due to the characteristics of nonneutral, cross-network externalities in bilateral market pricing, shared accommodation platform usually charges the service fee on one side and no frees on the other [23]. However, compared with hotel staff, most of the landlords attracted by shared accommodation platforms are untrained, nonprofessional training staff [24], and they also face ordinary consumers. There are many problems such as short development time, weak foundation, small development size, low participation enthusiasm in the development of the shared accommodation platform. Some scholars' studies have found that the profit has a greater influence on professional landlords in the operation process and the price level of their rooms is higher by comparing the operating conditions of the sellers operating a single room with those operating multiple rooms at the same time [25, 26]. There is a lack of research on how to change landlords' pricing strategies and develop reasonable pricing strategies.

As a successful sharing economy model, the shared accommodation platform is widely recognized in the world, and the reasonable operation strategy is the key to the development of the platform. Given the characteristics of sharing platforms, the products served by the sharing economy are different from traditional bilateral markets. In order to expand the size of the platform, many platforms consider using subsidies to regulate the balance between the supply and demand of bilateral users during their development phase, which has laid a good foundation for the successful operation of platforms, for example, Uber subsidized drivers and DiDi subsidized passengers. Subsidies can also be used as an incentive method to promote the increase of the number of participants in the development of shared accommodation platforms. It has been studied in the field of sharing economy platforms that the equilibrium market size obtained by subsidizing suppliers is larger than that obtained by subsidized customers [27]. As shared accommodation platforms adopt subsidies to expand the market size, subsidy policies need to be updated accordingly. Therefore, it is relevant and urgent to research the subsidy policies of shared accommodation platforms.

In order to promote the development of the accommodation industry in the context of big data and build a bilateral accommodation platform based on information technology, to make up for the insufficiency of previous research with two parties as game subjects, the behavioral strategies of all parties in the operation of the shared accommodation platform should be more comprehensive and systematic considered. This paper adopts evolutionary game theory to analyze the evolutionary mechanism and process among platform, landlord, and consumer under subsidy policy of the platform from a dynamic perspective and explores the influences of subsidy amounts, landlord service fee, consumer time cost, and the proportion of landlord's share in expenses on system stability. Finally, corresponding countermeasures are suggested for the subsidy mechanism of the platform. This paper answers the following three key questions: (1) How to determine the strategic conditions for shared accommodation platforms to conduct subsidies and the utilities obtained by consumers and landlords participating in shared accommodation. (2) How to achieve the equilibrium strategies of shared accommodation platforms, landlords, and consumers under platform subsidy mechanism. (3) To explore the factors affecting the equilibrium strategies.

## 2. Related Literature

Most of the research on shared accommodation focuses on the factors of willingness to participate of consumers and landlords and the factors influencing the price of shared accommodation.

Many scholars have explored consumers' willingness to participate based on different perspectives, and the studies have found that the material, energy-saving, psychological, and social interaction willingness promote consumers to participate in shared accommodation [28]. Extrinsic material benefits positively attract consumers to participate in shared accommodation platforms [29], and low-priced housing is the choice of most frugal consumers, which explains the attitude of consumers to use Airbnb [30]. Consumers tend to use rather than own products, which avoids the waste of social resources, and such behavior is expected to alleviate social problems such as excessive consumption and environmental pollution by reducing the cost of economic coordination [31]. Trust, familiarity, loyalty, authenticity, and hedonistic can also influence consumer participation in shared accommodation [32–34]. Social interactivity attracts consumers to share and helps to develop new social relationships during consumption [29, 35], enhancing social interaction stickiness [36]. Although the consumers' willingness to participate is affected by many factors, few studies consider the cross-network externalities between consumers and landlords.

Existing studies on landlords' willingness to participate are mostly centered on the economic value, functional value, security value, and trust value dimensions. The prerequisite for landlords to join the platform is the platform's sustainable development prospects, functional values of operational compatibility, and ability to respond to crises and solve problems [37]. By registering room resources online, the rental of idle rooms can be realized, and the participation enthusiasm of landlords can be stimulated by monetary value so that the landlords can directly obtain economic benefits [38, 39]. But landlords still have doubts about the safety and security of personal property in the platform [40]. Trust is the key factor to overcome uncertainty and reduce [41], so the importance of information security governance is particularly prominent [42]. It is necessary to clarify the influencing factors of landlords' participation in home-sharing so that the platform can formulate corresponding strategies to attract landlords to join.

The factors influencing the pricing of shared accommodation platforms are mainly the structure characteristics and accommodation facility features of the room, location characteristics, trust, and sociality [43–45]. The rental price is mainly determined by the landlord [43]. At the same time, the rental price is affected by the comment score and room quality, with higher pricing for high star rating listings [46]. The improvement in one room quality has the spillover effect on the prices of the other room [47]. There is a correlation between external factors and room rental prices. The internal facilities of the room and the supply of parking spaces have a positive impact on the room rental price, while instant booking has a negative effect on room rental prices [43, 48]. The authenticity of the photo has a significant impact on the rental price of the shared room; the more authentic the photo, the more purchase opportunities and economic benefits it brings [49]. However, few studies have discussed how to formulate reasonable strategies for sharing accommodation platforms to take into account the interests of all parties in sharing accommodation and achieve the optimal system utility.

The existing research methods on shared accommodation platforms are mainly to analyze the dynamic optimal allocation of idle rooms in the platform [50] and the game research between the participants of shared accommodation. However, most of the game models are studied from the perspective of two subjects, and there are few comprehensive studies on shared accommodation platforms, consumers, and landlords. The dynamic evolution law of the three stakeholders in the development process of the shared accommodation platform has not been deeply discussed; especially, the subsidy strategy of shared accommodation platforms has not been well explained. This study attempts to fill the gap by analyzing the impact of subsidy policies of shared accommodation platforms on the dynamic evolution of strategies among platform, consumer, and landlord, explores the evolution stage and influencing factors of platform subsidy implementation, and puts forward corresponding suggestions for platform development.

## 3. Materials and Methods

### 3.1. Problem Description

The shared accommodation platform can realize the sharing of information and resources and promote the rapid development of the shared accommodation industry. Currently, the shared accommodation industry is in the development stage, the platform's capacity is insufficient, the landlords are not dependent on the platform, and the consumers have limited access to information about room resources. Therefore, shared accommodation platform companies can give some subsidies for landlords and consumers to promote them to join the platform. The landlords share their idle rooms through the platform and obtain certain utilities, and consumers find satisfactory rooms through the shared accommodation platform. In this study, “platform” refers to the shared accommodation platform, “consumer” refers to the demander of the rental idle room, and “landlord” refers to the supplier of the rental idle room. The landlord (*L*) posts his or her idle room on the shared accommodation platform (*P*), and the consumer (*B*) rents it through the platform. The platform charges fees to the landlord and the consumer.

**Figure 1:**
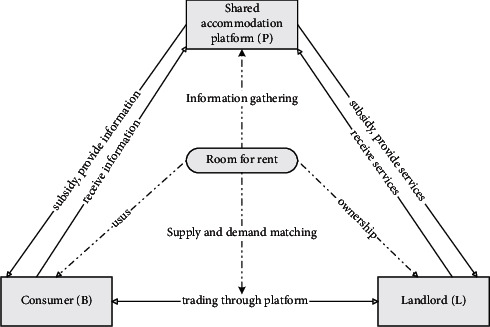
Relationship among shared accommodation platform, landlord, consumer, and room for rent.

The shared accommodation platform, landlords, and consumers are very important stakeholders in the development of the shared accommodation industry, and the three parties are connected through the rooms for rent, as shown in Figure 1.

### 3.2. Model Assumptions

In order to build the subsidy strategy model of a shared accommodation platform based on the evolutionary game in the context of big data, each party's strategy and the equilibrium point of the evolutionary game system are analyzed, and the following assumptions are made.

**Assumption 1 .**

In the network transaction system consisting of “shared accommodation platform-consumer-landlord,” each game participant is finite rational, and their information is asymmetric. In the game, the behaviors of the participants are random and interactional, which means that the participants cannot obtain the optimal strategies through one game but needs to go through trial and error and learn and improve their past strategies in the multiple rounds game. This means that each participant cannot obtain the optimal strategy through one game and needs to improve their strategies through continuous trial and error and learning in multiple rounds of games so as to make behavioral decisions that best match the current situation.

**Assumption 2 .**

tIn this paper, the goal of all parties is to maximize benefits under the subsidy policy of the platform. The platform determines whether to provide subsidies to consumers and landlords based on profit maximization; consumers decide whether to participate in sharing platform transactions based on their own needs to obtain the maximum utility of participating in sharing accommodation; landlords choose whether to join the platform based on the maximum net income of participating in the sharing platform. The maximum amounts of subsidy provided by the platform to landlords and consumers are, respectively, *S*_*L*_ and *S*_*B*_. The platform determines the intensity of subsidies according to the participation of landlords and consumers, and the proportion of subsidies provided by the platform to consumers and landlords is, respectively, *α* and *β*. Subsidies are given to landlords and consumers in the form of coupons or cash.

**Assumption 3 .**

In the process of operation, the sharing platform will charge corresponding fees to the landlords and consumers. *P*_*B*_ is the total cost paid by consumers to participate in shared accommodation, including the service fee charged by the platform and the fee charged by the landlord. The proportion of landlord's share in expenses paid by consumers is *ϕ.* The fee charged by the landlord is *ϕP*_*B*_, and the service fee charged by the platform is *P*_*B*_(1 − *ϕ*). *P*_*L*_ is the service fee charged by the platform to the landlord, and the service includes priority recommendation of room information, taking photos of the room, and providing smart locks.

**Assumption 4 .**

The strategy choice of shared accommodation platform is subsidy and nonsubsidy, the proportion of platform implementing subsidy is represented as *x*(0 ≤ *x* ≤ 1), and the proportion of implementing nonsubsidy is denoted as (1 − *x*); the strategy choices of consumer are participation and nonparticipation, the proportion of consumer implementing participation is represented as *y*(0 ≤ *y* ≤ 1), and the proportion of implementing nonparticipation is denoted as (1 − *y*); the strategy choices of the landlord are sharing and unsharing, the proportion of landlord implementing sharing is represented as *z*(0 ≤*z* ≤ 1), and the proportion of implementing unsharing is denoted as (1 − *z*), where *x*, *y*, *z* ∈ [0, 1].

**Assumption 5 .**

The purpose of adopting the subsidy is to enable consumers and landlords to participate in the shared accommodation platform, and the platform mainly obtains benefits by charging landlord service fees and the proportion of consumers paying room fees. Therefore, the initial subsidy of the platform needs to be more than the reputation benefit in order to better attract consumers and landlords to join the shared accommodation, that is, *αS*_*B*_ > *R*_*B*_ and *βS*_*L*_ > *R*_*L*_. Due to more contact with landlords on the sharing platform, the cross-network externalities utility of consumers received from participating in shared accommodation is more than the price paid by consumers, that is, *N*_*B*_ > *P*_*B*_.

### 3.3. Model Building

The parameters of this paper are described in Table 1.

According to the above assumptions, the shared accommodation platform, consumer, and landlord are the three main players in the game, and the game model benefit matrix is shown in Table 2.

### 3.4. Model Analysis

#### 3.4.1. Stability Analysis of Shared Accommodation Platform Strategies

The expected utility of shared platform selecting subsidy is *π*_*x*_, the expected utility of shared platform selecting nonsubsidy is *π*_1−*x*_, then the average expected utility of the shared platform is $\bar{\pi_{x}}$, and $\bar{\pi_{x}}=x\pi_{x}+\left(1-x\right)\pi_{1-x}$, which are defined as follows:

(1) $$\begin{array}{c} \pi_{x}=\text{yz}*\left[-C_{P}+P_{B}\left(1-\phi \right)+P_{L}+R_{B}+R_{L}-\left(\alpha S_{B}+\beta S_{L}\right)\right]+\left(1-y\right)\left(1-z\right)*\left(-C_{P}\right)+\left(1-y\right)z*\left(-C_{P}+R_{L}+P_{L}-\beta S_{L}\right)+y\left(1-z\right)*\left(-C_{P}+R_{B}-\alpha S_{B}\right).\\ \pi_{1-x}=\text{yz}*\left[-C_{P}+P_{B}\left(1-\phi \right)+P_{L}-H\right]+\left(1-y\right)\left(1-z\right)*\left(-C_{P}-H\right)+\left(1-y\right)z*\left(-C_{P}+P_{L}-H\right)+y\left(1-z\right)*\left(-C_{P}-H\right). \end{array}$$

According to the Malthusian dynamic equation, the replication dynamic equation of shared accommodation platform is obtained:

(2) $$\begin{array}{c} F\left(x\right)=\frac{\text{d}x}{\text{d}t}=x\left(1-x\right)*\left[y*\left(R_{B}-\alpha S_{B}\right)+z*\left(R_{L}-\beta S_{L}\right)+H\right],\\ F_{x}^{\prime }\left(x\right)=\frac{\partial F\left(x\right)}{\partial x}=\left(1-2x\right)*\left[y*\left(R_{B}-\alpha S_{B}\right)+z*\left(R_{L}-\beta S_{L}\right)+H\right]. \end{array}$$

According to the stability theorem of the replication dynamic equation, the evolutionary stability strategy of the shared platform must be satisfied with the conditions that *F*(*x*)=0 and *F*_*x*_′(*x*)＜0. And the threshold of shared platform strategy transformation is *y*^*∗*^=−((*z∗*(*R*_*L*_ − *βS*_*L*_)+*H*)/(*R*_*B*_ − *αS*_*B*_)).

**Proposition 1 .**

In the evolution process, when 0 < *y* < *y*^*∗*^ < 1, the stabilization strategy of shared accommodation platform is subsidy. When 0 < *y* < *y*^*∗*^ < 1, the stabilization strategy of shared accommodation platform is nonsubsidy.

**Proof**

Assume *G*(*y*)=*y∗*(*R*_*B*_ − *αS*_*B*_)+*z∗*(*R*_*L*_ − *βS*_*L*_)+*H*; when *R*_*B*_ − *αS*_*B*_ < 0, *G(y)* is considered to be a decreasing function of *y*. When *y* >  *y*^*∗*^, *G(y)* < 0, *F*(*x*)*|*_*x*=0_=0, and *F*_*x*_′(*x*)*|*_*x*=0_ < 0, *x* = 0 is the stabilization point; that is, the nonsubsidy of the shared accommodation platform is the stabilization strategy; when *y* <  *y*^*∗*^, *G(y)* > 0, *F*(*x*)*|*_*x*=1_=0, and *F*_*x*_′(*x*)*|*_*x*=1_ < 0, *x* = 1 is the stabilization point; that is, the subsidy of the shared accommodation platform is a stabilization strategy; when *y*= *y*^*∗*^, *x* is stable in the interval from 0 to 1; that is, regardless of the initial proportion of the strategy selected by the shared accommodation platform, the platform will not change the strategy.

Proposition 1 shows that when the reputational benefits of platform subsidy to consumers are less than the subsidy amount of the platform to consumers, the reduction of consumer participation will change the stability strategy of the shared accommodation platform from nonsubsidy to subsidy. Similarly, the increase of consumer participation will change the stability strategy of the shared accommodation platform from subsidy to nonsubsidy. Therefore, the participation strategy of consumers is crucial to the implementation of platform subsidy, and it is also the basis of platform subsidy strategy.

**Figure 2:**
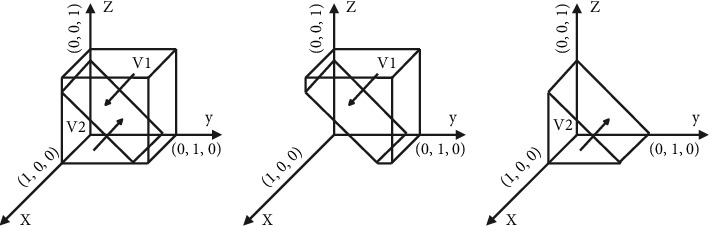
Phase diagram of strategy evolution of shared accommodation platforms. (a) *y*=−((*z∗*(*R*_*L*_ − *βS*_*L*_)+*H*)/(*R*_*B*_ − *αS*_*B*_)), (b) *y* > −((*z∗*(*R*_*L*_ − *βS*_*L*_)+*H*)/(*R*_*B*_ − *αS*_*B*_)), and (c) *y* < −((*z∗*(*R*_*L*_ − *βS*_*L*_)+*H*)/(*R*_*B*_ − *αS*_*B*_)).

The phase diagram of the strategic evolution of the shared accommodation platform is shown in Figure 2.

Figure 2 shows that the volume of *V*_1_ represents the proportion of subsidy of the shared accommodation platform, and the volume of *V*_2_ represents the proportion of nonsubsidy of the shared accommodation platform, which is calculated as follows:

(3) $$\begin{array}{c} V_{1}=\int_{0}^{1}\int_{-H/R_{L}-\beta S_{L}}^{1}-\frac{z*\left(R_{L}-\beta S_{L}\right)+H}{R_{B}-\alpha S_{B}}\text{d}z\text{d}x=\frac{{\left(H+a_{1}\right)}^{2}}{2a_{1}*b_{1}}, \end{array}$$

(4) $$\begin{array}{c} V_{2}=1-V_{1}, \end{array}$$

where *a*_1_=*R*_*L*_ − *βS*_*L*_ and *b*_1_=*R*_*B*_ − *α*_1_*S*_*B*_.

**Corollary 1 .**

The proportion of shared accommodation platform subsidy is positively correlated with the reputation benefits of the platform subsidizing and the opportunity loss of the platform nonsubsidizing and negatively correlated with the subsidy amount of the platform subsidizing to consumers and landlords.

**Proof**

According to the formula (4), when *H* > *βS*_*L*_ − *R*_*L*_; *H* > *αS*_*B*_ − *R*_*B*_, the first-order partial derivative of each element is obtained as follows: (∂*V*_1_/∂*R*_*L*_) > 0, (∂*V*_1_/∂*R*_*B*_) > 0, (∂*V*_1_/∂*H*) > 0, (∂*V*_1_/∂*β*  *S*_*L*_) < 0, and (∂*V*_1_/∂*α*  *S*_*B*_) < 0 (see Appendix A). Therefore, the increase of *R*_*L*_, *R*_*B*_, and *H* and the decrease of *βS*_*L*_ and *αS*_*B*_ can increase the proportion of subsidizing for shared accommodation platforms.

Corollary 1 shows that, with the increase in the reputation benefits of the platform due to subsidies to consumers and landlords and the opportunity cost of the platform nonsubsidy, the sharing accommodation platform is more inclined to adopt the subsidy strategy. As the amount of subsidies provided by the platform to landlords and consumers increases, the shared accommodation platform is more inclined to adopt a nonsubsidy strategy.

#### 3.4.2. Stability Analysis of Consumer Strategies

The expected utility of the consumer choosing to participate is *π*_*y*_, the expected utility of the consumer choosing nonparticipating is *π*_1−*y*_, then the average expected utility of the consumer is $\bar{\pi_{y}}$, and $\bar{\pi_{y}}=y\pi_{y}+\left(1-y\right)\pi_{1-y}$, which are defined as follows:

(5) $$\begin{array}{c} \pi_{y}=xz*\left(-P_{B}-C_{B}+U_{B}+\alpha S_{B}+N_{B}\right)+\left(1-x\right)\left(1-z\right)*\left(-C_{B}\right)+x\left(1-z\right)*\left(\alpha S_{B}-C_{B}\right)+\left(1-x\right)z*\left(-P_{B}-C_{B}+U_{B}+N_{B}\right),\\ \pi_{1-y}=xz*\left(-B\right)+\left(1-x\right)\left(1-z\right)*\left(-B\right)\;+x\left(1-z\right)*\left(-B\right)+\left(1-x\right)z*\left(-B\right). \end{array}$$

According to the Malthusian dynamic equation, the replication dynamic equation of consumer is obtained:

(6) $$\begin{array}{c} F\left(y\right)=\frac{\text{d}y}{\text{d}t}=y\left(1-y\right)*\left[z*\left(-P_{B}+N_{B}\right)+x*\alpha S_{B}+U_{B}-C_{B}+B\right],\\ F_{y}^{\prime }\left(y\right)=\frac{\partial F\left(y\right)}{\partial y}=\left(1-2y\right)*\left[z*\left(-P_{B}+N_{B}\right)+x*\alpha S_{B}+U_{B}-C_{B}+B\right]. \end{array}$$

According to the stability theorem of the replication dynamic equation, the evolutionary stability strategy of the consumer must be satisfied with the conditions that *F*(*y*)=0 and *F*_*y*_′(*y*) < 0. And the threshold of the consumer strategy transformation is *z*^*∗*^=(−*x∗αS*_*B*_+*C*_*B*_ − *U*_*B*_ − *B*)/(*N*_*B*_ − *P*_*B*_).

**Proposition 2 .**

In the evolution process, when 0 < *z*^*∗*^ < *z* < 1, the stabilization strategy of consumer is participation. When 0 < *z*^*∗*^ < *z* < 1, the stabilization strategy of consumer is nonparticipation.

**Proof**

Assume *G*(*z*)=*z∗*(−*P*_*B*_+*N*_*B*_)+*x∗αS*_*B*_+*U*_*B*_ − *C*_*B*_+*B*; when *N*_*B*_ > *P*_*B*_*C*_*B*_ − *U*_*B*_ − *B* > *αS*_*B*_, *G(z)* is considered to be an increasing function of *z*. When *z* > *z*^*∗*^, *G(z)*  >  0, *F*(*y*)*|*_*y*=1_=0, and *F*_*y*_′(*y*)*|*_*y*=1_ < 0, then *y*  =  1 is the stabilization point; that is, consumer participation is the stabilization strategy; when *z* <  *z*^*∗*^, *G(z)*  <  0, *F*(*y*)*|*_*y*=0_=0, and *F*_*y*_′(*y*)*|*_*y*=0_ < 0, *y*  =  0 is the stabilization point; that is, the nonparticipation of the consumer is the stabilization strategy; when *z*=*z*^*∗*^, *y* is stable in the interval from 0 to 1; that is, regardless of the initial proportion of the strategy selected by the consumer, the consumer will not change the strategy.

Proposition 2 shows that the strategic choice of the landlord will affect the consumer's choice. When the cross-network externalization utility gained by the consumer with the landlord on the shared platform is more than the fees paid by the consumer participating in the shared accommodation platform, with the increase of the landlord's share, the consumer's stabilization strategy will be changed from nonparticipation to participation. Similarly, the decrease in landlord's share will change the consumer's stabilization strategy from participation to nonparticipation. Therefore, to promote consumer participation in shared accommodation, the platform should take proactive measures to allow more landlords to share their rooms and improve the network cross-externalities utility between consumers and landlords to make it habitual for consumers to participate in shared accommodation.

**Figure 3:**
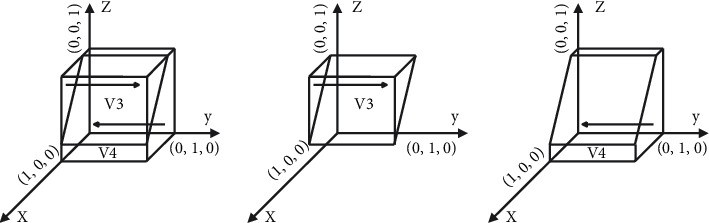
Phase diagram of the evolution of the consumer's strategy. (a) *z*=((−*x∗αS*_*B*_+*C*_*B*_ − *U*_*B*_ − *B*)/(*N*_*B*_ − *P*_*B*_)), (b) *z* > ((−*x∗αS*_*B*_+*C*_*B*_ − *U*_*B*_ − *B*)/(*N*_*B*_ − *P*_*B*_)), and (c) *z* < ((−*x∗αS*_*B*_+*C*_*B*_ − *U*_*B*_ − *B*)/(*N*_*B*_ − *P*_*B*_)).

The phase diagram of the strategic evolution of the consumer is shown in Figure 3.

Figure 3 shows that the volume of *V*_*3*_ represents the proportion of consumers participating in shared accommodation, and the volume of *V*_*4*_ represents the proportion of consumers nonparticipation in shared accommodation, which is calculated as follows:

(7) $$\begin{array}{c} V_{3}=\int_{0}^{1}\int_{-C_{B}+U_{B}+B/\alpha S_{B}}^{1}\frac{-x*\alpha S_{B}+C_{B}-U_{B}-B}{N_{B}-P_{B}}\text{d}x\text{d}y=\frac{2a_{2}*b_{2}-a_{2}^{2}-b_{2}^{2}}{2a_{2}*c_{2}}, \end{array}$$

(8) $$\begin{array}{c} V_{4}=1-V_{3}, \end{array}$$

where *a*_2_=*αS*_*B*_, *b*_2_=*C*_*B*_ − *U*_*B*_ − *B*, and *c*_2_=*N*_*B*_ − *P*_*B*_.

**Corollary 2 .**

The proportion of consumer participation in shared accommodation is positively correlated with the subsidy amount given to consumers by the platform, the utility of platform service for the consumer, the fees paid by consumers for renting rooms through offline channels, and the cross-network externalization utility gained by consumers with landlords on the shared platform. The proportion of consumer participation in shared accommodation is negatively correlated with the consumers' time cost and the fees paid by consumers participating in shared accommodation platforms.

**Proof**

According to the formula (8), when *C*_*B*_ > *U*_*B*_+*B*; *C*_*B*_ − *U*_*B*_ − *B* > *N*_*B*_ − *P*_*B*_, the first-order partial derivative of each element is obtained as follows: (∂*V*_3_/∂*α*  *S*_*B*_) > 0, (∂*V*_3_/∂*C*_*B*_) < 0, (∂*V*_3_/∂*U*_*B*_) > 0, (∂*V*_3_/∂*B*) > 0, (∂*V*_3_/∂*N*_*B*_) > 0, and (∂*V*_3_/∂*P*_*B*_) < 0 (see Appendix B). Therefore, the increase of *αS*_*B*_, *U*_*B*_, *B*, and *N*_*B*_ and the decrease of *C*_*B*_ and *P*_*B*_ can increase the proportion of consumer participation in shared accommodation.

Corollary 2 shows that, with the increase of the subsidies amount for consumers gained from the platform, the utility of platform services for the consumer, the fees paid for obtaining rooms through offline channels, and the utility of cross-network externalities of landlords to consumers, the consumer is more inclined to adopt the participation strategy. As the time cost of consumers and the payment price of consumers' participation in shared accommodation increase, the consumer is more inclined to adopt the nonparticipation strategy.

#### 3.4.3. Stability Analysis of Landlord Strategies

The expected utility of the landlord choosing to share the rooms is *π*_*z*_, the expected utility of the landlord choosing to unshare the rooms is *π*_1−*z*_, then the average expected utility of landlord is $\bar{\pi_{z}}$, and $\bar{\pi_{z}}=z\pi_{z}+\left(1-z\right)\pi_{1-z}$, which are defined as follows:

(9) $$\begin{array}{c} \pi_{z}=xy*\left(\phi P_{B}+\beta S_{L}+N_{L}+U_{L}-C_{L}-P_{L}\right)+\left(1-x\right)\left(1-y\right)*\left(\begin{array}{c} U_{L}-C_{L}-P_{L} \end{array}\right)+x\left(1-y\right)*\left(\beta S_{L}+U_{L}-C_{L}-P_{L}\right)+\left(1-x\right)y*\left[\phi P_{B}+N_{L}+U_{L}-C_{L}-P_{L}\right],\\ \pi_{1-z}=xy*A+\left(1-x\right)y*A+x\left(1-y\right)*A+\left(1-x\right)\left(1-y\right)*A. \end{array}$$

According to the Malthusian dynamic equation, the replication dynamic equation of landlord is obtained:

(10) $$\begin{array}{c} \;F\left(z\right)=\frac{\text{d}z}{\text{d}t}=z\left(1-z\right)*\left[y*\left(\phi P_{B}+N_{L}\right)+x*\beta S_{L}+U_{L}-C_{L}-\;P_{L}-A\right],\\ F_{z}^{\prime }\left(z\right)=\frac{\partial F\left(z\right)}{\partial z}=\left(1-2z\right)*\left[y*\left(\phi P_{B}+N_{L}\right)+x*\beta S_{L}+U_{L}-C_{L}-\;P_{L}-A\right]. \end{array}$$

According to the stability theorem of the replication dynamic equation, the evolutionary stability strategy of the landlord must be satisfied with the conditions that *F*(*z*)=0 and *F*_*z*_′(*z*) < 0. And the threshold of the consumer strategy transformation is *x*^*∗*^=(−*y∗*(*ϕP*_*B*_+*N*_*L*_) − *U*_*L*_+*C*_*L*_+*P*_*L*_+*A*)/*βS*_*L*_.

**Proposition 3 .**

In the evolution process, when 0 < *x*^*∗*^ < *x* < 1, the stabilization strategy of landlord is sharing. When 0 < *x*^*∗*^ < *x* < 1, the stabilization strategy of landlord is unsharing.

**Proof**

Assume *G*(*x*)=*y∗*(*ϕP*_*B*_+*N*_*L*_)+*x∗βS*_*L*_+*U*_*L*_ − *C*_*L*_ −  *P*_*L*_ − *A*; when *C*_*L*_+ *P*_*L*_+*A* − *U*_*L*_ > *ϕP*_*B*_+*N*_*L*_, *G(x)* is considered to be an increasing function of *x*. When *x* > *x*^*∗*^, *G(x)* > 0, *F*(*z*)*|*_*z*=1_=0, and *F*_*z*_′(*z*)*|*_*z*=1_ < 0, *z* = 1 is the stabilization point; that is, the share of the landlord is the stabilization strategy; when *x* <  *x*^*∗*^, *G(x)* < 0, *F*(*z*)*|*_*z*=0_=0, and *F*_*z*_′(*z*)*|*_*z*=0_ < 0, *z* = 0 is the stabilization point; that is, the unshare of the landlord is the stabilization strategy; when *x*=*x*^*∗*^, *z* is in an evolutionary stabilization state; that is, regardless of the initial proportion of the strategy selected by the landlord, the landlord will not change the strategy.

Proposition 3 shows that the strategy choice of shared accommodation platforms will affect the choice of the landlord. The increase in the proportion of the platform subsidy will change the landlord's stabilization strategy from nonsharing to sharing. Similarly, the reduction of the platform subsidy proportion will change the landlord's stabilization strategy from sharing to nonsharing. Therefore, platform subsidy is a powerful means to promote landlords to share.

**Figure 4:**
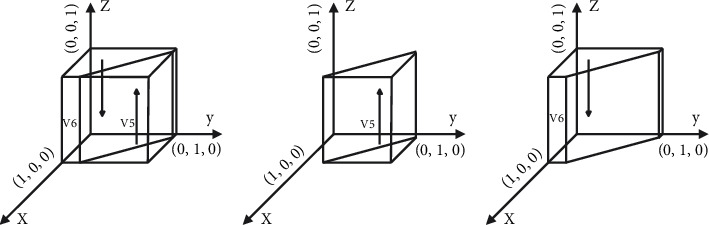
Phase diagram of evolution strategy of the landlord. (a) *x*=((*C*_*L*_+ *P*_*L*_+*A* − *y∗*(*ϕP*_*B*_+*N*_*L*_) − *U*_*L*_)/*βS*_*L*_), (b) *x* > ((*C*_*L*_+ *P*_*L*_+*A* − *y∗*(*ϕP*_*B*_+*N*_*L*_) − *U*_*L*_)/*βS*_*L*_), and (c) *x* < ((*C*_*L*_+ *P*_*L*_+*A* − *y∗*(*ϕP*_*B*_+*N*_*L*_) − *U*_*L*_)/*βS*_*L*_).

The phase diagram of the strategic evolution of the landlord is shown in Figure 4.

Figure 4 shows that the volume of *V*_5_ represents the proportion of the landlord sharing the rooms, and the volume of *V*_6_ represents the proportion of the landlord unsharing the rooms, which is calculated as follows:

(11) $$\begin{array}{c} V_{5}=\int_{0}^{1}\int_{-U_{L}+C_{L}+\;P_{L}+A/\phi P_{B}+N_{L}}^{1}\frac{-y*\left(\phi P_{B}+N_{L}\right)-U_{L}+C_{L}+\;P_{L}+A}{\beta S_{L}}\text{d}y\text{d}z=\frac{2a_{3}*b_{3}-a_{3}^{2}-b_{3}^{2}}{2a_{3}*c_{3}}, \end{array}$$

(12) $$\begin{array}{c} V_{6}=1-V_{5}, \end{array}$$

where *a*_3_=*βS*_*L*_, *b*_3_=−*U*_*L*_+*C*_*L*_+*P*_*L*_+*A*, and *c*_3_=*ϕP*_*B*_+*N*_*L*_.

**Corollary 3 .**

The proportion of the landlord sharing rooms in the shared accommodation platform is positively correlated with the subsidy amount of the platform to landlords, the utility of platform service for the landlord, the fees paid by consumers participating in shared accommodation platforms, and the cross-network externalities utility gained by landlords with consumers on the shared platform. The proportion of the landlord sharing rooms is negatively correlated with the income by the landlord with unsharing room in the platform, the cost of room and service provided by the landlord, and the fees charged to landlords for the platform services.

**Proof**

According to the formula (11), when *C*_*L*_+ *P*_*L*_+*A* > *U*_*L*_ and *C*_*L*_+ *P*_*L*_+*A* − *U*_*L*_ > *βS*_*L*_, the first-order partial derivative of each element is obtained: (∂*V*_5_/∂*β*  *S*_*L*_) > 0, (∂*V*_5_/∂*C*_*L*_) < 0, (∂*V*_5_/∂*P*_*L*_) < 0, (∂*V*_5_/∂*A*) < 0, (∂*V*_5_/∂*U*_*L*_) > 0, (∂*V*_5_/∂*ϕ*  *P*_*B*_) > 0, and (∂*V*_5_/∂*N*_*L*_) > 0 (see Appendix C). Therefore, the increase of *βS*_*L*_, *U*_*L*_, *ϕP*_*B*_, and *N*_*L*_ and the decrease of *A*, *C*_*L*_, and *P*_*L*_ can increase the proportion of the landlord participating in the shared rooms.

Corollary 3 shows that, with the increase of subsidy amount of the platform to landlords, the utility of platform service for the landlord, the fees paid by consumers participating in shared accommodation platform, and the utility of cross-network externalities of consumers to landlords, the landlord is more inclined to adopt the share strategy. As the increase of the income by the landlord with unsharing room, the cost of room and service provided by the landlord, and the fees charged to landlords for the platform services, the landlord is more inclined to adopt the nonshare strategy.

## 4. Results and Discussion

### 4.1. Strategy Portfolio Stability Analysis

In the dynamic system of the shared accommodation platform, consumers, and landlords, the stability of the strategic combination of the three-party evolutionary game can be referred to Liapunov's nonlinear function stability discriminant method. When all eigenvalues of the Jacobi matrix are less than zero, the equilibrium point must be asymptotically stabilization; when the first-order bias of the matrix is positive or semipositive and one or two of the eigenvalues of the Jacobi matrix are greater than zero, the equilibrium point is the unstable point or the saddle point. Therefore, in the three-party evolutionary game, the stability of the eight pure strategy equilibrium will be analyzed in this paper. That is, *E*1(0, 0, 0), *E*2(1, 0, 0), *E*3(0, 1, 0), *E*4(0, 0, 1), *E*5(1, 1, 0), *E*6(1, 0, 1), *E*7(0, 1, 1), and *E*8(1, 1, 1).

According to the dynamic equations of each game subject, the Jacobian matrix is obtained as follows:

(13) $$\begin{array}{c} J=\left[\begin{array}{ccc} F_{x}^{\prime }\left(x\right) & F_{y}^{\prime }\left(x\right) & F_{z}^{\prime }\left(x\right)\\ F_{x}^{\prime }\left(y\right) & F_{y}^{\prime }\left(y\right) & F_{z}^{\prime }\left(y\right)\\ F_{x}^{\prime }\left(z\right) & F_{y}^{\prime }\left(z\right) & F_{z}^{\prime }\left(z\right) \end{array}\right], \end{array}$$

where the values of the Jacobi matrix are shown in Appendix D and the Jacobian matrix of the equilibrium point is shown in Appendix E.

According to the Jacobian matrix, the stability analysis of the equilibrium point of the three-party evolutionary game can be obtained, and it is shown in Table 3.

When condition I is satisfied, that is *αS*_*B*_+*U*_*B*_ − *C*_*B*_ < *B*; *βS*_*L*_+*U*_*L*_ − *C*_*L*_ − *P*_*L*_ < *A*, the dynamic system has a stable point, that is, *E*2(1, 0, 0). The consumer chooses not to participate in shared accommodation when the sum of the subsidy amount and the net utility gained by consumers from the platform is less than the fees paid by consumers for renting rooms through offline channels. The landlord chooses to unshare the rooms when the sum of the subsidy amount and the net utility gained by the landlord from the platform is less than the income by the landlord with unsharing room in the platform. The stable strategy of sharing accommodation platforms is the subsidy. This may happen in the early stage of the development of shared accommodation. When shared accommodation platform is just getting started, landlords and consumers do not know enough about shared accommodation. The platform adopts a subsidy strategy to attract landlords and consumers. When the shared accommodation platform has just started, the platform adopts the subsidy strategy to attract landlords and consumers to join in because of their insufficient awareness of shared accommodation.

When condition II is satisfied, that is *αS*_*B*_+*βS*_*L*_ − *R*_*B*_ − *R*_*L*_ < *H*; *P*_*B*_+*C*_*B*_ − *B* < *N*_*B*_+*αS*_*B*_+*U*_*B*_ ; *C*_*L*_+*P*_*L*_+*A* < *ϕP*_*B*_+*βS*_*L*_+*N*_*L*_+*U*_*L*_, the dynamic system has a stable point, that is, *E*8(1, 1, 1). The shared accommodation platform chooses the subsidy strategy when the difference between the total subsidy amount and the reputational benefits of the platform is less than the opportunity loss of platform nonsubsidizing. The consumer chooses to participate in shared accommodation when the difference between the time cost and the fees of consumers in the shared accommodation platform and the fees paid by the consumer for renting rooms through offline channels is less than the utility and benefit that the consumer obtains on the sharing platform. The landlord chooses to share the rooms when the utility and benefit that the landlord obtains on the sharing platform are more than the sum of the cost paid by the landlord on the sharing platform and the income by the landlord with unsharing room in the platform. This may happen during the rapid development of shared accommodation platform. The platform develops appropriate subsidy strategies, such as issuing coupons and cash rewards, to promote more consumers to participate in shared accommodation and landlords to share the rooms, which make landlords and consumers form the habits of participating in sharing accommodation.

When condition III is satisfied, that is *αS*_*B*_+*βS*_*L*_ − *R*_*B*_ − *R*_*L*_ > *H*; *P*_*B*_+*C*_*B*_ − *B* < *N*_*B*_+*U*_*B*_; *C*_*L*_+*P*_*L*_+*A* < *ϕP*_*B*_+*N*_*L*_+*U*_*L*_, the replicated dynamic system has a stable point, that is, *E*7(0, 1, 1). The shared accommodation platform chooses the subsidy strategy when the difference between the total subsidy amount and the reputational benefits of the platform is more than the opportunity loss of platform nonsubsidizing. The consumer chooses to participate in shared accommodation when the difference between the time cost and the fees of consumers in the shared accommodation platform and the fees paid by the consumer for renting rooms through offline channels is less the utility gained by consumers on the shared platform. The landlord chooses to share the rooms when the utility and benefit that the landlord obtains on the sharing platform are more than the sum of the cost paid by the landlord on the sharing platform and the income by the landlord with unsharing room in the platform. This may happen in the mature stage of shared accommodation platform development. The landlords and the consumers have already understood shared accommodation, and the platform has developed a series of sound policies to push the information and serve the landlords and consumers. The platforms can make considerable gains by choosing nonsubsidizing.

### 4.2. Simulation Analysis

In order to test the reliability of the model and to more intuitively observe the evolution trajectory of stakeholders and their sensitivity to parameters, this paper uses MATLAB2016 to simulate the evolution stability strategy and related parameters.

#### 4.2.1. Model Testing

**Figure 5:**
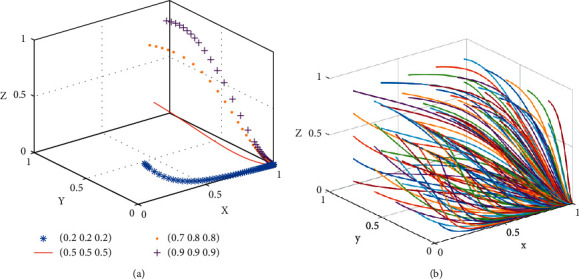
Evolutionary equilibrium diagram at the point *E*2(1, 0, 0).

When condition I is satisfied, the simulation parameters are set as follows: *C*_*P*_ = 5, *C*_*B*_ = 11, *C*_*L*_ = 3, *R*_*B*_ = *R*_*L*_ = 2, *S*_*B*_ = *S*_*L*_ = 5, *P*_*L*_ = *P*_*B*_ = 5, *α* = 0.1, *β* = 0.3, *ϕ* = 0.7, *A* = *H* = 3, *B* = 6, *N*_*B*_ = 6, *N*_*L*_ = 2, *U*_*B*_ = 1, and *U*_*L*_ = 3. Selecting four different initial strategies, all four groups of initial strategies all evolve towards *E*2(1, 0, 0), and the results of simulation and evolution with time are shown in Figure 5.

It can be seen from Figure 5 that when shared accommodation platform is in the early stage of development, the consumer and landlord do not have a certain understanding of the sharing accommodation platform. For the needs of market expansion, shared accommodation platform subsidizes consumers and landlords to promote them to join the shared accommodation platform.

**Figure 6:**
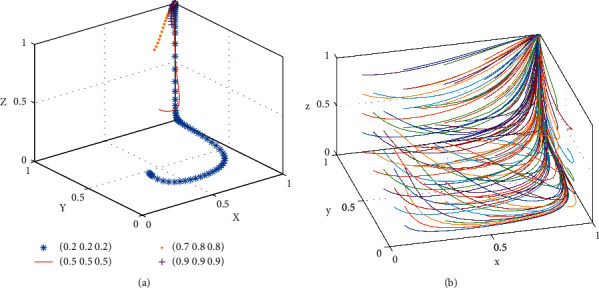
Evolutionary equilibrium diagram at the point *E*8(1, 1, 1).

When condition II is satisfied, the simulation parameters are set as follows: *C*_*B*_ = 8, *P*_*L*_ = 3, *α* = 0.6, and *β* = 0.5, and other parameters are the same with the equilibrium point E2(1, 0, 0). Selecting four different initial strategies, all four groups of initial strategies all evolve towards *E*8(1, 1, 1), and the results of simulation and evolution with time are shown in Figure 6.

It can be seen from Figure 6 that, with the reduction of the time cost of the consumer participating in shared accommodation and the increase of the service utility and the subsidies to consumers by the platform, the actual cost of consumer participating in shared accommodation is less than the cost through offline channels. Therefore, the consumer will choose to participate in shared accommodation platform. With the increase of the subsidy to the landlord and reduction of the service fee for the landlord to participate in the shared accommodation platform, the landlord's revenue from shared rooms is more than the loss of the landlord's inventory for nonparticipating in sharing rooms. The landlord will adopt the strategy to actively join the shared accommodation platform to share rooms. In the end, the platform, consumers, and landlords will tend to adopt the positive strategies.

**Figure 7:**
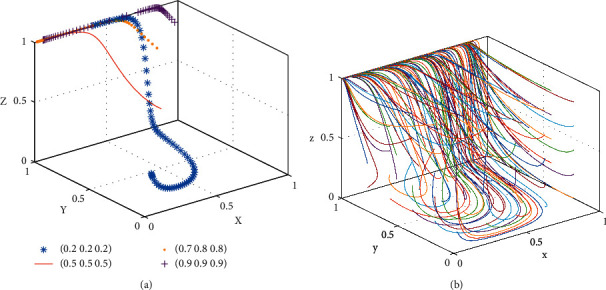
Evolutionary equilibrium diagram at the point *E*7(0, 1, 1).

When condition III is satisfied, the simulation parameters are set as follows: *C*_*L*_ = 1, *P*_*B*_ = 3, *ϕ* = 0.9, and *H* = 1, and other parameters are the same with the equilibrium point *E*8(1, 1, 1). Selecting four different initial strategies, all four groups of initial strategies all evolve towards *E*7(0, 1, 1), and the results of simulation and evolution with time are shown in Figure 7.

It can be seen from Figure 7 that, with the reduction of the time cost for consumers and the fees of the landlords to participate in shared accommodation, the consumer and landlord are promoted to participate in the shared accommodation platform. When the consumers and landlords become accustomed to participating in the shared accommodation platform to reduce the opportunity loss of platform nonsubsidizing, the platform can appropriately reduce the subsidies and the cost paid by consumers to participate in shared accommodation and increase the share of the revenue for landlords. Consumers and landlords will still choose to participate in shared accommodation. In the mature stage of the development of the shared accommodation platform, the platform can set the reasonable price for consumers and the preferential proportion of the landlord's share in expenses under the nonsubsidy policy, the landlords, and consumers can continue to participate in the shared accommodation to maximize the interests of all three parties.

#### 4.2.2. The Influence of Different Subjects' Initial Willingness to Participate

**Figure 8:**
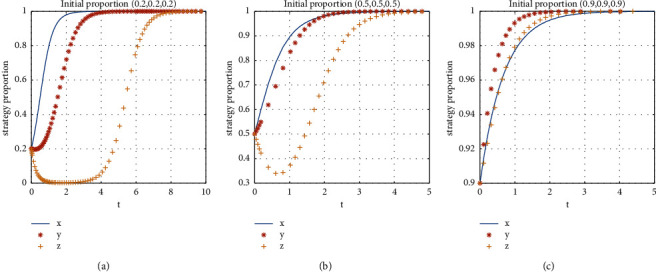
Influence of different subjects' initial willingness to participate on the evolutionary.

The initial proportions of the strategies of the shared accommodation platform, the consumer, and the landlord are set as follows: (0.2, 0.2, 0.2), (0.5, 0.5, 0.5), and (0.9, 0.9, 0.9). The stability of the system evolution is shown in Figure 8.

It can be seen from Figure 8 that, under the parameter conditions of the evolutionary stability strategy *E*8(1, 1, 1), the behavioral strategies of the shared accommodation platform, consumers, and landlords finally evolve into the strategies of “subsidy,” “participation,” and “share” with the initial proportions of the strategies of (0.2, 0.2, 0.2), (0.5, 0.5, 0.5), and (0.9, 0.9, 0.9). This indicates that the stable state of the system is independent of the initial proportions of the strategies. Further analysis reveals that, in the case of low initial willingness to participate, landlords choose the unsharing strategy because they can obtain the rental income of the rooms in other ways. When the platform's subsidy for consumers and landlords increases and the time cost of consumers and landlords' service fees to participate in shared accommodation platform decreases, the consumer chooses to participate, and landlord will choose to share. When the initial willingness of the platform, consumer, and landlord is high, all three participants will continue to evolve steadily to the strategies of “subsidy,” “participation,” and “share.” The closer the initial proportion is to the proportion of an evolutionary stable state, the shorter the time for evolution to reach the stable state is.

#### 4.2.3. The Influence of the Proportion of Subsidies Provided by the Platform to Consumers

**Figure 9:**
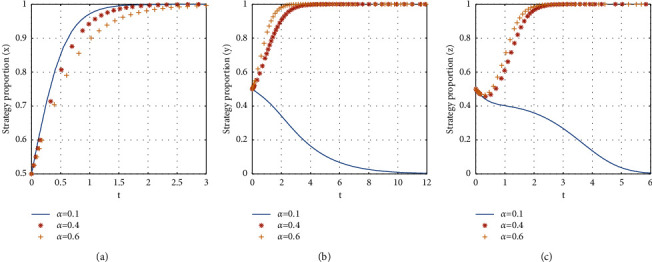
The influence of the proportion of subsidies to the consumer on evolutionary stabilization strategies of subject behaviors.

If *α* = {0.1, 0.4, 0.6}, the stabilization strategy of each participant is obtained as shown in Figure 9.

From Figure 9, it can be seen that, with the increase of the proportion of subsidy to the consumer, the evolutionary stability strategy transitions from *E*2(1, 0, 0) to *E*8(1, 1, 1). The critical value of the proportion of subsidy to consumers is between 0.4 and 0.6. When the proportion of subsidies to the consumer is larger than the critical value, the evolutionary stability strategy of the three-party game converges to *E*8(1, 1, 1), and the larger the proportion of consumer subsidies is, the faster the evolutionary stability strategy converges. When the proportion of consumer subsidies is less than the critical value, consumer chooses not to participate in the shared accommodation platform, the landlords choose to unshare the rooms, and the time for the landlord to reach stability is faster than that for consumers. Therefore, the platform can increase the proportion of subsidy for consumers to promote the participation of the consumer and the landlord.

#### 4.2.4. The Influence of the Proportion of Subsidies Provided by the Platform to Landlords

**Figure 10:**
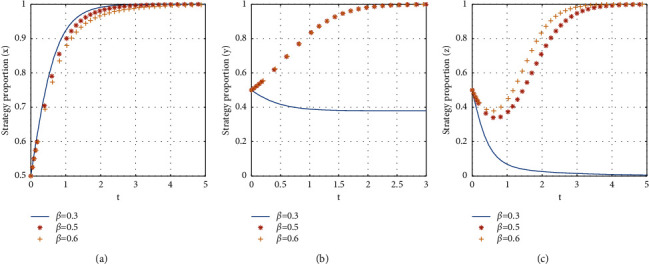
The influence of the proportion of subsidies to the landlord on evolutionary stabilization strategies of subject behaviors.

If *β* = {0.3, 0.5, 0.6}, the stabilization strategy of each participant is obtained as shown in Figure 10.

From Figure 10, it can be seen that, with the increase of the proportion of the subsidy to the landlord, the evolutionary stability strategy transitions from *E*2(1, 0, 0) to *E*8(1, 1, 1). The critical value of the proportion of the subsidy to the landlord is between 0.5 and 0.6. When the proportion of subsidies to the landlord is larger than the critical value, the evolutionary stability strategy of the three-party game converges to *E*8(1, 1, 1), and the larger the proportion of landlord subsidies is, the faster the evolutionary stability strategy converges. When the proportion of subsidies to the landlord is less than the critical value, the consumer chooses not to participate in the shared accommodation, the landlord chooses to unshare the rooms, and the time for the landlord to reach stability is slower than that for the consumer to reach stability. It is a benefit for landlords to participate in the shared accommodation platform to increase the subsidies to landlords. Therefore, in the early stage of the platform's development, choosing the more share of subsidy to the landlord can promote consumers and landlords to join the shared accommodation platform more quickly.

#### 4.2.5. The Influence of the Fees Charged to Landlords for the Platform Services

**Figure 11:**
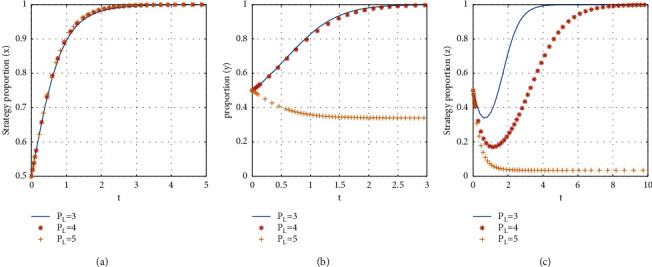
The influence of the fees to the landlord for the platform services on evolutionary stabilization strategies of subject behaviors.

If *P*_*L*_ = {3, 4, 5}, the stabilization strategy of each participant is obtained as shown in Figure 11.

From Figure 11, it can be seen that, with the decrease of the fees charged to landlords for the platform services, the evolutionary stability strategy transitions from *E*2(1, 0, 0) to *E*8(1, 1, 1). The critical value of the fees charged to landlords for the platform services is between 4 and 5. When the fees to the landlord for the platform services are less than the critical value, the evolutionary stability strategy of the three-party game converges to *E*8(1, 1, 1), and the less the fees to the landlord for the platform services are, the faster the evolutionary stability strategy converges. When the fees to the landlord for the platform services are more than the critical value, the consumer chooses not to participate in shared accommodation, the landlord chooses to unshare the rooms, and the time for the landlord to reach stability is slower than that for the consumer to reach stability. Therefore, in the early stage of the platform's development, charging fewer fees to landlords for the platform services can promote consumers and landlords to join the shared accommodation platform more quickly.

#### 4.2.6. The Influence of the Time Costs of the Consumer

**Figure 12:**
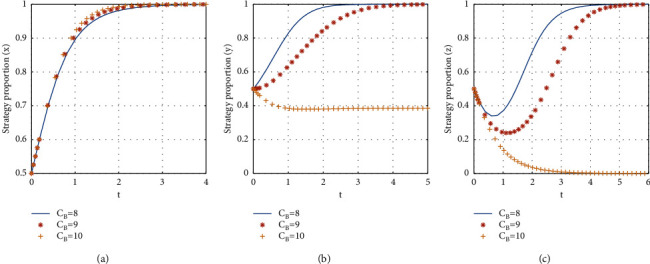
The influence of the consumer's time costs on evolutionary stabilization strategies of subject behaviors.

If *C*_*B*_ = {8, 9, 10}, the stabilization strategy of each participant is obtained as shown in Figure 12.

From Figure 12, it can be seen that, with the decrease of the consumer's time costs, the evolutionary stability strategy transitions from *E*2(1, 0, 0) to *E*8(1, 1, 1). The critical value of the time cost of the consumer to participate in the shared accommodation platform is between 9 and 10. When the time costs of the consumer are less than the critical value, the evolutionary stability strategy of the three-party game converges to *E*8(1, 1, 1), and the less the consumer's time costs are, the faster the evolutionary stability strategy converges. When the consumer's time costs are more than the critical value, the consumer chooses not to participate in the shared accommodation, the landlord chooses to unshare the rooms, and the time for the landlord to reach stability is slower than the time for the consumer to reach stability. Therefore, in the early stage of the platform's development, decreasing the time costs of the consumer can promote consumers and landlords to join the shared accommodation platform more quickly.

#### 4.2.7. The Influence of the Proportion of Landlord's Share in Expenses Paid by Consumers

**Figure 13:**
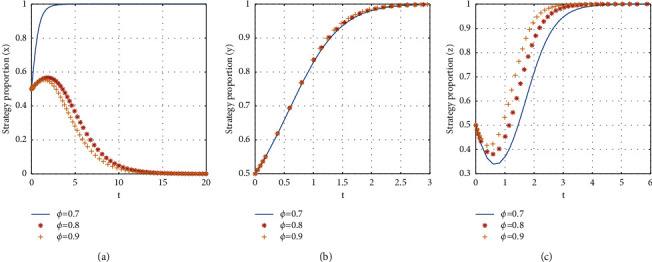
The influence of the proportion of landlord's share in expenses on evolutionary stabilization strategies of subject behaviors.

If *ϕ* = {0.7, 0.8, 0.9}, the stabilization strategy of each participant is obtained as shown in Figure 13.

From Figure 13, it can be seen that, with the increase of the proportion of landlords' share in expenses, the evolutionary stability strategy transitions from *E*8(1, 1, 1) to *E*7(0, 1, 1). The critical value of the proportion of the landlord's share in expenses paid by consumers is between 0.7 and 0.8. When the proportion of the landlord's share in expenses is less than the critical value, the evolutionary stability strategy of the three-party game converges to *E*8(1, 1, 1). When the value of the proportion of the landlord's share in expenses is larger than the critical value, the shared accommodation platform chooses the nonsubsidy strategy. And the larger the proportion of the landlord's share in expenses is, the faster the evolutionary stability strategy of the shared accommodation platform converges. Therefore, in the rapid development of shared accommodation platforms, increasing the proportion of landlords' share in expenses to meet the interests of landlords can stabilize the choice of the landlord participating in the shared accommodation platform. When the net income of landlords and consumers reaches a certain level with the increase of the scale, the shared accommodation platform can achieve the optimization of system benefits with nonsubsidizing.

#### 4.2.8. The Influence of the Opportunity Loss of Platform Nonsubsidizing

**Figure 14:**
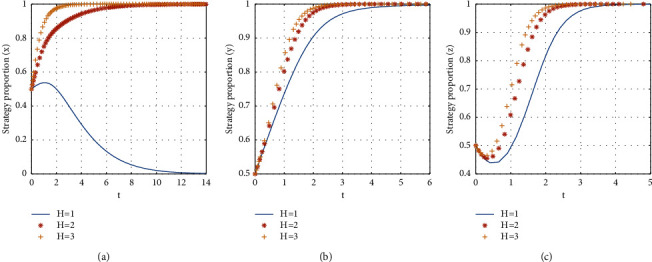
The influence of the platform's opportunity loss on evolutionary stabilization strategies of subject behaviors.

If *H* = {1, 2, 3}, the stabilization strategy of each participant is obtained as shown in Figure 14.

From Figure 14, it can be seen that, with the decrease of the opportunity loss of platform nonsubsidizing, the evolutionary stability strategy transitions from *E*8(1, 1, 1) to *E*7(0, 1, 1). The critical value of the opportunity loss of platform nonsubsidizing is between 1 and 2. When the opportunity loss of platform nonsubsidizing is more than the critical value, the evolutionary stability strategy of the three-party game converges to *E*8(1, 1, 1). And when the opportunity loss of platform nonsubsidizing is less than the critical value, the shared accommodation platform chooses the nonsubsidy strategy. The less the opportunity loss of platform nonsubsidizing is, the faster the evolutionary stability strategy of the shared accommodation platform converges. Therefore, in the rapid development of shared accommodation platforms, the decrease of the opportunity loss of platform nonsubsidizing indicates that the consumer and landlord have formed the habit of participating in shared accommodation platform, and the strategy of the platform gradually evolved to the nonsubsidy.

## 5. Conclusions

Internet and big data technologies provide the technological impetus for the development of new economic formats, and effective governance has become the key to the success of platforms [51, 52]. Platform-based companies aim to maximize the revenue of the platform, and gaining economic benefits is also the important intention of each subject to participate in collaborative consumption [53]. Aiming at the subsidy strategy of shared accommodation platform, an evolutionary game model was established, the system stability was analyzed and simulated, and the main conclusions of this paper are as follows:

1. The evolutionary stability strategy of the shared accommodation platform based on subsidizing can go through three stages. At the early stage of platform establishment, the shared accommodation platform attracts consumers and landlords to join through subsidizing due to the need for market expansion. At this time, consumers refuse to join the shared accommodation platform for the high time cost, landlords refuse to join for the high service fee paid, and the system will stabilize on the strategic combination of *E*2(subsidy, nonparticipation, unsharing). In this state, consumers and landlords have a preliminary understanding and experience of the sharing accommodation platform. When the difference between the time cost and the fees of consumers in the shared accommodation platform and the fees paid by the consumer for renting rooms through offline channels is less than the utility and benefit that the consumer obtains on the sharing platform, the consumer chooses to join the shared accommodation platform. When the utility and benefit that the landlord obtains on the sharing platform are more than the sum of the cost paid by the landlord on the sharing platform and the income by the landlord with unsharing room in the platform, the landlord chooses to join in the shared accommodation platform. At this time, the system stabilizes on the strategic combination of *E*8(subsidy, participation, sharing). Due to the influx of landlords and consumers, the shared accommodation platform is expanding rapidly. When the difference between the total subsidy amount and the reputational benefits of the platform is more than the opportunity loss of platform nonsubsidizing, the platform chooses the nonsubsidy strategy. At this time, it is necessary to meet that the utility of consumers and landlords is more than their costs, and the system will stabilize on the strategic combination of *E*7(nonsubsidy, participation, sharing). The shared accommodation platform no longer needs subsidy to attract landlords and consumers and relies on quality services to achieve good operation of the platform, and the shared accommodation platform reaches the mature stage of development. The dynamic change analysis of the strategies in these three stages makes up for the deficiency of the subsidy strategy of the accommodation platform in the existing research.
2. When the strategy combination of the three parties transitions from *E*2(subsidy, nonparticipation, unshare) to *E*8(subsidy, participation, share), it can be achieved by increasing the subsidies to consumers and landlords and reducing consumers' time costs and service fees charged to the landlord. These measures to increase the profits of consumers and landlords and to reduce their losses are all aimed at attracting consumers and landlords to join the shared accommodation platform. This is consistent with the conclusion proposed by Guo et al. [27] that the bilateral platform of the sharing economy can adopt the subsidy strategy.
3. When the strategy combination of the three parties transitions from *E*8(subsidize, participate, share) to *E*7(nonsubsidy, participation, share), it can be achieved by reducing the opportunity loss of platform nonsubsidy and increasing the proportion of the landlord's share in expenses paid by consumers. Therefore, as consumers and landlords form the habit of participating in shared accommodation platform, landlords and consumers can maintain good profits for the three parties through normal transactions so that the loss of the platform nonsubsidy is reduced, and the strategy of the platform is gradually changed from subsidy to nonsubsidy.

In this paper, the evolutionary game model of three parties in the shared accommodation platform is established, and it is found that the subsidy strategy of the shared accommodation platform can encourage consumers and landlords to participate in shared accommodation. By adjusting the subsidies to customers and landlords, the time cost of consumers, the service fees of landlords, the proportion of landlord's share of consumers' fees, and the loss of the platform's nonsubsidy, the evolutionary equilibrium of the game strategy can be changed. This study makes up for the deficiency of three-party game analysis in the existing literature and does in-depth research on sharing economy by using the game theory.

In order to activate the sharing market and further develop the sharing economy platform, the following suggestions are put forward:

1. The subsidies can be used by the platform to attract landlords and consumers. When the platform is in the initial development stage, the subsidies are an effective way to expand the number of platform users. However, the specific subsidy amount must be considered in combination with the use costs of consumers and landlords, and the platform will try to give an attractive subsidy amount. If the construction of the platform goes smoothly, the subsidy policy can also be adjusted to realize the normal operation of the shared accommodation platform.
2. In the development process of the platform, by fully excavating the massive and scattered rooms, the diversity of shared accommodation products is ensured to provide consumers with diverse accommodation options. The platform can use information integration to optimize the platform interface for reducing the time for consumers to choose room information. On this basis, the standardization of shared accommodation services is promoted to further improve the quality of products and services.
3. The service utility of the platform to the landlords can be increased to maintain the stability of the landlord's rooms in order to ensure the supply for customers. The platform can reduce the service fee charged to the landlord and increase services quality for landlords. For example, Airbnb provides photo services to landlords, smart door locks, face-brushing check-in, and so on. The platform can also establish a quick communication channel between landlords and customers to help landlords reduce communication costs.

This study establishes a three-party game evolution model based on subsidy policies. Due to the limitations of model assumptions, under the condition of asymmetric information and bounded rationality, the benefits of shared accommodation platforms, consumers, and landlords are maximized in subsidy policies, and the possibility of unsuccessful matching between consumers and landlords in the shared accommodation platform is not considered. Therefore, in order to improve the operating mechanism of the shared accommodation platform, it will be the further research direction to consider the role of factors affecting participants' participation in the strategic evolution, such as the possibility of matching between consumers and landlords, as well as the location and facility differences of rooms.

## Figures and Tables

**Table 1:** Parameter description.

Parameter	Description
*C*_*P*_	Platform operating costs
*R*_*B*_	Reputational benefits of platform subsidizing to consumers
*R*_*L*_	Reputational benefits of platform subsidizing to landlords
*S*_*B*_	Maximum subsidy amount of the platform to consumers
*Α*	The proportion of subsidies provided by the platform to consumers, *α* ∈ [0,1]
*S*_*L*_	Maximum subsidy amount of the platform to landlords
*B*	The proportion of subsidies provided by the platform to landlords, *β* ∈ [0,1]
*H*	Opportunity loss of platform nonsubsidizing
*P*_*L*_	Fees charged to landlords for the platform services
*P*_*B*_	Fees paid by consumers participating in shared accommodation platforms
*Φ*	The proportion of landlord's share in expenses paid by consumers, ∅∈[0,1]
*N*_*B*_	Cross-network externalization utility gained by consumers with landlords on the shared platform
*C*_*B*_	Time cost of consumers
*B*	Fees paid by consumers for renting room through offline channels
*U*_*B*_	The utility of platform service for the consumer
*N*_*L*_	Cross-network externalities utility gained by landlords with consumers on shared platform
*U*_*L*_	The utility of platform service for the landlord
*C*_*L*_	The cost of room and service provided by the landlord
*A*	Income by the landlord with unsharing room in the platform

**Table 2:** Game model benefit matrix of shared accommodation platform, consumer, and landlord.

Strategy choice		Shared accommodation platform	
Landlord	Consumer	Subsidy (*x*)	Nonsubsidy (1 − *x*)
Share (*z*)	Participation (*y*)	*U*_*B*_ * − C*_*P*_ * + P*_*B*_ *(1 − ϕ) + P*_*L*_ *+* *R*_*B*_* + R*_*L*_* − αS*_*B*_* − βS*_*L*_*−P*_*B*_* − C*_*B*_* + U*_*B*_* + αS*_*B*_* + N*_*B*_*ϕP*_*B*_* + βS*_*L*_* + N*_*L*_* + U*_*L*_* − C*_*L*_* − P*_*L*_	*−C*_*P*_ * + P*_*B*_ *(1 − ϕ) + P*_*L*_ * − H–P*_*B*_ * – C*_*B*_ * + U*_*B*_ * + N*_*B*_ *ϕP*_*B*_ * + N*_*L*_ * + U*_*L*_ * − C*_*L*_ * − P*_*L*_
	Nonparticipation (1* − y*)	*−C*_*P*_ * + P*_*L*_ * + R*_*L*_ * − βS*_*L*_ *−BβS*_*L*_ * + U*_*L*_ * − C*_*L*_ * − P*_*L*_	*−C*_*P*_ * − H + P*_*L*_ *−BU*_*L*_ * − C*_*L*_ * − P*_*L*_

Unshare (1 − *z*)	Participation (*y*)	*−C*_*P*_ * + R*_*B*_ * − αS*_*B*_ *αS*_*B*_ * − C*_*B*_ *A*	*−C*_*P*_ * − H−C*_*B*_ *A*
	Nonparticipation (1* − y*)	*−C*_*P*_ *−BA*	*−C*_*P*_ * − H−BA*

**Table 3:** Stability analysis of equilibrium point of evolutionary game.

Equilibrium point	Eigenvalue symbol	Stability of the equilibrium point	Condition
*E*1(0, 0, 0)	(+, +, −)	Saddle point	\
*E*2(1, 0, 0)	(−, *X*, *X*)	ESS	I
*E*3(0, 1, 0)	(+, +, −)	Saddle point	\
*E*4(0, 0, 1)	(+, −, +)	Saddle point	\
*E*5(1, 1, 0)	(+, +, −)	Unstable point	\
*E*6(1, 0, 1)	(−, *X*, +)	Unstable point	\
*E*7(0, 1, 1)	(*X*, *X*, *X*)	ESS	III
*E*8(1, 1, 1)	(*X*, *X*, *X*)	ESS	II

*Note. X* means uncertainty of symbol, and ESS means the evolutionarily stable strategy. Condition I: *αS*_*B*_+*U*_*B*_ − *C*_*B*_ < *B*; *βS*_*L*_+*U*_*L*_ − *C*_*L*_ − *P*_*L*_ < *A*. Condition II: *αS*_*B*_+*βS*_*L*_ − *R*_*B*_ − *R*_*L*_ < *H*; *P*_*B*_+*C*_*B*_ − *B* < *N*_*B*_+*αS*_*B*_+*U*_*B*_; *C*_*L*_+*P*_*L*_+*A* < *ϕP*_*B*_+*βS*_*L*_+*N*_*L*_+*U*_*L*_. Condition III: *αS*_*B*_+*βS*_*L*_ − *R*_*B*_ − *R*_*L*_ > *H*; *P*_*B*_+*C*_*B*_ − *B* < *N*_*B*_+*U*_*B*_; *C*_*L*_+*P*_*L*_+*A* < *ϕP*_*B*_+*N*_*L*_+*U*_*L*_.

## Data Availability

The data used to support the findings of the study are available from the corresponding author upon request.

## Appendix

### A. Supplementary Proof of Corollary 1

The proportion of subsidy strategy of shared accommodation platform is *V*_*1*_, and the first-order partial derivative of each element is proved as follows:

(A.1) $$\begin{array}{c} V_{1}=\frac{{\left(H+a_{1}\right)}^{2}}{2a_{1}*b_{1}}, \end{array}$$

where *a*_1_=*R*_*L*_ − *βS*_*L*_ and *b*_1_=*R*_*B*_ − *α*_1_*S*_*B*_.

When *b*_1_ < 0 and *H*+*a*_1_ > 0,

(A.2) $$\begin{array}{c} \frac{\partial V_{1}}{\partial R_{L}}={\left(\frac{{\left(H+a_{1}\right)}^{2}}{2a_{1}*b_{1}}\right)}^{\prime }=-\frac{H\left(H+a_{1}\right)}{a_{1}^{2}*b_{1}}>0,\\ \frac{\partial V_{1}}{\partial R_{B}}={\left(\frac{{\left(H+a_{1}\right)}^{2}}{2a_{1}*b_{1}}\right)}^{\prime }=-\frac{{\left(H+a_{1}\right)}^{2}}{2a_{1}*b_{1}^{2}}>0,\\ \frac{\partial V_{1}}{\partial H}={\left(\frac{{\left(H+a_{1}\right)}^{2}}{2a_{1}*b_{1}}\right)}^{\prime }=\frac{H+a_{1}}{a_{1}*b_{1}}>0,\\ \frac{\partial V_{1}}{\partial \beta \;S_{L}}={\left(\frac{{\left(H+a_{1}\right)}^{2}}{2a_{1}*b_{1}}\right)}^{\prime }=-\frac{\partial V_{1}}{\partial R_{L}}<0,\\ \frac{\partial V_{1}}{\partial \alpha \;S_{B}}={\left(\frac{{\left(H+a_{1}\right)}^{2}}{2a_{1}*b_{1}}\right)}^{\prime }=-\frac{\partial V_{1}}{\partial R_{B}}<0. \end{array}$$

### B. Supplementary Proof of Corollary 2

The proportion of participating strategy of the consumer is *V*_3_, and the first-order partial derivative of each element is proved as follows:

(B.1) $$\begin{array}{c} V_{3}=\frac{2a_{2}*b_{2}-a_{2}^{2}-b_{2}^{2}}{2a_{2}*c_{2}}, \end{array}$$

where *a*_2_=*αS*_*B*_, *b*_2_=*C*_*B*_ − *U*_*B*_ − *B*, and *c*_2_=*N*_*B*_ − *P*_*B*_.

(B.2) $$\begin{array}{c} \frac{\partial V_{3}}{\partial \alpha \;S_{B}}={\left(\frac{2a_{2}*b_{2}-a_{2}^{2}-b_{2}^{2}}{2a_{2}*c_{2}}\right)}^{\prime }=\frac{b_{2}^{2}-2b_{2}*c_{2}-a_{2}^{2}}{2a_{2}^{2}*c_{2}}>0,\\ \frac{\partial V_{3}}{\partial C_{B}}={\left(\frac{2a_{2}*b_{2}-a_{2}^{2}-b_{2}^{2}}{2a_{2}*c_{2}}\right)}^{\prime }=\frac{c_{2}-b_{2}}{a_{2}*c_{2}}<0,\\ \frac{\partial V_{3}}{\partial U_{B}}=\frac{\partial V_{3}}{\partial B}={\left(\frac{2a_{2}*b_{2}-a_{2}^{2}-b_{2}^{2}}{2a_{2}*c_{2}}\right)}^{\prime }=-\frac{\partial V_{3}}{\partial C_{B}}>0,\\ \frac{\partial V_{3}}{\partial N_{B}}={\left(\frac{2a_{2}*b_{2}-a_{2}^{2}-b_{2}^{2}}{2a_{2}*c_{2}}\right)}^{\prime }=\frac{a_{2}^{2}+b_{2}^{2}}{2a_{2}*c_{2}^{2}}>0,\\ \frac{\partial V_{3}}{\partial P_{B}}={\left(\frac{2a_{2}*b_{2}-a_{2}^{2}-b_{2}^{2}}{2a_{2}*c_{2}}\right)}^{\prime }=-\frac{\partial V_{3}}{\partial N_{B}}<0. \end{array}$$

### C. Supplementary Proof of Corollary 3

The proportion of share strategy of the landlord is *V*_5_, and the first-order partial derivative of each element is proved as follows:

(C.1) $$\begin{array}{c} V_{5}=\frac{2a_{3}*b_{3}-a_{3}^{2}-b_{3}^{2}}{2a_{3}*c_{3}}, \end{array}$$

where *a*_3_=*βS*_*L*_ , *b*_3_=−*U*_*L*_+*C*_*L*_+ *P*_*L*_+*A*, and *c*_3_=*ϕP*_*B*_+*N*_*L*_.

(C.2) $$\begin{array}{c} \frac{\partial V_{5}}{\partial \beta \;S_{L}}={\left[\frac{2a_{3}*b_{3}-a_{3}^{2}-b_{3}^{2}}{2a_{3}*c_{3}}\right]}^{\prime }=\frac{b_{3}^{2}-2b_{3}*c_{3}-a_{3}^{2}}{2a_{3}^{2}*c_{3}}>0,\\ \frac{\partial V_{5}}{\partial C_{L}}={\left[\frac{2a_{3}*b_{3}-a_{3}^{2}-b_{3}^{2}}{2a_{3}*c_{3}}\right]}^{\prime }=\frac{c_{3}-b_{3}}{a_{3}*c_{3}}<0,\\ \frac{\partial V_{5}}{\partial P_{L}}=\frac{\partial V_{5}}{\partial A}={\left[\frac{2a_{3}*b_{3}-a_{3}^{2}-b_{3}^{2}}{2a_{3}*c_{3}}\right]}^{\prime }=\frac{\partial V_{5}}{\partial C_{L}}<0,\\ \frac{\partial V_{5}}{\partial U_{L}}={\left[\frac{2a_{3}*b_{3}-a_{3}^{2}-b_{3}^{2}}{2a_{3}*c_{3}}\right]}^{\prime }=-\frac{\partial V_{5}}{\partial C_{L}}>0,\\ \frac{\partial V_{5}}{\partial \phi \;P_{B}}={\left[\frac{2a_{3}*b_{3}-a_{3}^{2}-b_{3}^{2}}{2a_{3}*c_{3}}\right]}^{\prime }=\frac{a_{3}^{2}+b_{3}^{2}}{2a_{3}*c_{3}^{2}}>0,\\ \frac{\partial V_{5}}{\partial N_{L}}={\frac{2a_{3}*b_{3}-a_{3}^{2}-b_{3}^{2}}{2a_{3}*c_{3}}}^{\prime }=\frac{\partial V_{5}}{\partial \phi \;P_{B}}>0. \end{array}$$

### D. Jacobian Matrix of Replicator Dynamic System

The elements of the Jacobian matrix is as follows:

(D.1) $$\begin{array}{c} F\left(x\right)=\frac{\text{d}x}{\text{d}t}=x\left(1-x\right)*\left[y*\left(R_{B}-\alpha S_{B}\right)+z*\left(R_{L}-\beta S_{L}\right)+H\right],\\ F_{x}^{\prime }\left(x\right)=\frac{\partial F\left(x\right)}{\partial x}=\left(1-2x\right)*\left[y*\left(R_{B}-\alpha S_{B}\right)+z*\left(R_{L}-\beta S_{L}\right)+H\right],\\ F_{y}^{\prime }\left(x\right)=\frac{\partial F\left(x\right)}{\partial y}=x\left(1-x\right)*\left(R_{B}-\alpha S_{B}\right),\\ F_{z}^{\prime }\left(x\right)=\frac{\partial F\left(x\right)}{\partial z}=x\left(1-x\right)*\left(R_{L}-\beta S_{L}\right),\\ F\left(y\right)=\frac{\text{d}y}{\text{d}t}=y\left(1-y\right)*\left[z*\left(-P_{B}+N_{B}\right)+x*\alpha S_{B}+U_{B}-C_{B}+B\right],\\ F_{x}^{\prime }\left(y\right)=\frac{\partial F\left(y\right)}{\partial x}=y\left(1-y\right)*\alpha S_{B},\\ F_{y}^{\prime }\left(y\right)=\frac{\partial F\left(y\right)}{\partial y}=\left(1-2y\right)*\left[z*\left(-P_{B}+N_{B}\right)+x*\alpha S_{B}+U_{B}-C_{B}+B\right],\\ F_{z}^{\prime }\left(y\right)=\frac{\partial F\left(y\right)}{\partial z}=y\left(1-y\right)*\left(-P_{B}+N_{B}\right),\\ F\left(z\right)=\frac{\text{d}z}{\text{d}t}=z\left(1-z\right)*\left[y*\left(\phi P_{B}+N_{L}\right)+x*\beta S_{L}+U_{L}-C_{L}-\;P_{L}-A\right],\\ F_{x}^{\prime }\left(z\right)=\frac{\partial F\left(z\right)}{\partial x}=z\left(1-z\right)*\beta S_{L},\\ F_{y}^{\prime }\left(z\right)=\frac{\partial F\left(z\right)}{\partial y}=z\left(1-z\right)*\left(\phi P_{B}+N_{L}\right),\\ F_{z}^{\prime }\left(z\right)=\frac{\partial F\left(z\right)}{\partial z}=\left(1-2z\right)*\left[y*\left(\phi P_{B}+N_{L}\right)+x*\beta S_{L}+U_{L}-C_{L}-\;P_{L}-A\right]. \end{array}$$

### E. Jacobian Matrix of Equilibrium Points of Replicator Dynamic System

The Jacobian matrix of the equilibrium point *E*1(0, 0, 0) is as follows:

(E.1) $$\begin{array}{c} J_{1}=\left[\begin{array}{ccc} H & 0 & 0\\ 0 & U_{B}-C_{B}+B & 0\\ 0 & 0 & U_{L}-C_{L}-\;P_{L}-A \end{array}\right]. \end{array}$$

The Jacobian matrix of the equilibrium point *E*2(1, 0, 0) is as follows:

(E.2) $$\begin{array}{c} J_{2}=\left[\begin{array}{ccc} -H & 0 & 0\\ 0 & \alpha S_{B}+U_{B}-C_{B}+B & 0\\ 0 & 0 & \beta S_{L}+U_{L}-C_{L}-\;P_{L}-A \end{array}\right]. \end{array}$$

The Jacobian matrix of the equilibrium point *E*3(0, 1, 0) is as follows:

(E.3) $$\begin{array}{c} J_{3}=\left[\begin{array}{ccc} R_{B}-\alpha S_{B}+H & 0 & 0\\ 0 & -U_{B}+C_{B}-B & 0\\ 0 & 0 & \phi P_{B}+N_{L}+U_{L}-C_{L}-\;P_{L}-A \end{array}\right]. \end{array}$$

The Jacobian matrix of the equilibrium point *E*4(0, 0, 1) is as follows:

(E.4) $$\begin{array}{c} J_{4}=\left[\begin{array}{ccc} R_{L}-\beta S_{L}+H & 0 & 0\\ 0 & U_{B}-C_{B}+B-P_{B}+N_{B} & 0\\ 0 & 0 & -U_{L}+C_{L}+\;P_{L}+A \end{array}\right]. \end{array}$$

The Jacobian matrix of the equilibrium point E5(1, 1, 0) is as follows:

(E.5) $$\begin{array}{c} J_{5}=\left[\begin{array}{ccc} -R_{B}+\alpha S_{B}-H & 0 & 0\\ 0 & -\alpha S_{B}-U_{B}+C_{B}-B & 0\\ 0 & 0 & \phi P_{B}+N_{L}+\beta S_{L}+U_{L}-C_{L}-\;P_{L}-A \end{array}\right]. \end{array}$$

The Jacobian matrix of the equilibrium point *E*6(1, 0, 1) is as follows:

(E.6) $$\begin{array}{c} J_{6}=\left[\begin{array}{ccc} -R_{L}+\beta S_{L}-H & 0 & 0\\ 0 & -P_{B}+N_{B}+\alpha S_{B}+U_{B}-C_{B}+B & 0\\ 0 & 0 & -\beta S_{L}-U_{L}+C_{L}+\;P_{L}+A \end{array}\right]. \end{array}$$

The Jacobian matrix of the equilibrium point *E*7(0, 1, 1) is as follows:

(E.7) $$\begin{array}{c} J_{7}=\left[\begin{array}{ccc} R_{B}-\alpha S_{B}+R_{L}-\beta S_{L}+H & 0 & 0\\ 0 & P_{B}-N_{B}-U_{B}+C_{B}-B & 0\\ 0 & 0 & -\phi P_{B}-N_{L}-U_{L}+C_{L}+P_{L}+A \end{array}\right]. \end{array}$$

The Jacobian matrix of the equilibrium point *E*8(1, 1, 1) is as follows:

(E.8) $$\begin{array}{c} J_{8}=\left[\begin{array}{ccc} -R_{B}+\alpha S_{B}-R_{L}+\beta S_{L}-H & 0 & 0\\ 0 & P_{B}-N_{B}-\alpha S_{B}-U_{B}+C_{B}-B & 0\\ 0 & 0 & -\phi P_{B}-N_{L}-\beta S_{L}-U_{L}+C_{L}+\;P_{L}+A \end{array}\right]. \end{array}$$

## References

[1] Botsman R. (2011). *What’s Mine Is Yours the Rise of Collaborative Consumption*.

[2] Belk R. (2007). Why not share rather than own?. *The Annals of the American Academy of Political and Social Science*.

[3] Albinsson P. A., Perera B. Y. (2012). Alternative marketplaces in the 21st century: building community through sharing events. *Journal of Consumer Behaviour*.

[4] Zhou K., He L. Y., Zhang Y. W. (2020). A review of literature on the concept, impacts, and spatial interactions of sharing short-term rental platform. *Progress in Geography*.

[5] Grybaitė V., Stankevičienė J. (2018). An empirical analysis of factors affecting sharing economy growth. *Oeconomia Copernicana*.

[6] Netter S., Pedersen E., Lüdeke-Freund F. (2019). Sharing economy revisited: towards a new framework for understanding sharing models. *Journal of Cleaner Production*.

[7] Garcia-Ayllon S. (2018). Urban transformations as an indicator of unsustainability in the P2P mass tourism phenomenon: the Airbnb case in Spain through three case studies. *Sustainability*.

[8] Ranjbari M., Morales-Alonso G., Carrasco-Gallego R. (2018). Conceptualizing the sharing economy through presenting a comprehensive framework. *Sustainability*.

[9] Hu S., Yang X., Wang Q. (2020). Research review on sharing accommodation at home and abroad. *Tourism Science*.

[10] Yang Y., Tan K. P.-S., Li X. (2019). Antecedents and consequences of home-sharing stays: evidence from a nationwide household tourism survey. *Tourism Management*.

[11] Long F., Liu J., Zhu H., Li T. (2019). Spatial distribution and influencing factors of homestays in Yangtze River Delta. *Geography Research*.

[12] Zhang H., Lu L., Zhang D., Yu H., Zhang X. (2019). Temporal and spatial distribution characteristics and causes of homestays around Moganshan. *Geography Research*.

[13] He L. (2016). Analysis of the business model of online short rent enterprises - take Piggy short rent as an example. *Modern Business*.

[14] Reid J., Wilson K., Anderson K. E., Maguire C. P. J. (2015). Older inpatients’ room preference: single versus shared accommodation. *Age and Ageing*.

[15] Ling C., Zhang Z. (2014). Research on the development path of ‘sharing economy’ in China - take online short rent as an example. *Modern Management Science*.

[16] Wang Y., Yang L. (2017). Inspiration of Airbnb business model to China Online short rent Industry. *Modern Business*.

[17] Zhao C. (2016). Sharing economy: an analysis of the competition structure of China online short rent industry based on five forces model. *Economic Research Guide*.

[18] Xiao Y. (2015). How to solve ‘acclimatization’ in online short rent. *Legal Person*.

[19] Li L., Su J. (2019). Shared accommodation: the change and influence of the relationship between subject and object. *Tourism Forum*.

[20] Bae S. J., Lee H., Suh E.-K., Suh K.-S. (2017). Shared experience in pretrip and experience sharing in posttrip: a survey of Airbnb users. *Information & Management*.

[21] Guttentag D. (2015). Airbnb: disruptive innovation and the rise of an informal tourism accommodation sector. *Current Issues in Tourism*.

[22] Lee C. K. H., Tse Y. K. (2021). Improving peer-to-peer accommodation service based on text analytics. *Industrial Management & Data Systems*.

[23] Zhang X., Sui R., Dan B., Guan Z. (2021). Shared accommodation: the change and influence of the relationship between subject and object. *Computers & Industrial Engineering*.

[24] Li J., Moreno A., Zhang D. J. (2015). Agent behavior in the sharing economy: evidence from Airbnb. *SSRN Electronic Journal*.

[25] Kwok L., Xie K. L (2019). Pricing strategies on Airbnb: are multi-unit hosts revenue pros?. *International Journal of Hospitality Management*.

[26] Gibbs C., Guttentag D., Gretzel U., Morton J., Goodwill A. (2018). Pricing in the sharing economy: a hedonic pricing model applied to Airbnb listings. *Journal of Travel & Tourism Marketing*.

[27] Guo Y., Li X., Zeng X. (2019). Platform competition in the sharing economy: understanding how ride-hailing services influence new car purchases. *Journal of Management Information Systems*.

[28] Yu H., Tian L., Jiang G., Yun C. (2018). Sharing economy: theory and research agenda. *Nankai Business Review*.

[29] Guttentag D., Smith S., Potwarka L., Havitz M. (2018). Why tourists choose Airbnb: a motivation-based segmentation study. *Journal of Travel Research*.

[30] So K. K. F., Oh H., Min S. (2018). Motivations and constraints of Airbnb consumers: findings from a mixed-methods approach. *Tourism Management*.

[31] Hamari J., Sjöklint M., Ukkonen A. (2016). The sharing economy: why people participate in collaborative consumption. *Journal of the Association for Information Science and Technology*.

[32] Mhlmann M. (2015). Collaborative consumption: determinants of satisfaction and the likelihood of using a sharing economy option again. *Journal of Consumer Behaviour*.

[33] Konak S., Özhasar Y. (2020). The reasons of foreign tourists choose of Airbnb when they has visited İstanbul. *International Journal of Contemporary Tourism Research*.

[34] Wu J., Zeng M., Xie K. (2017). Chinese travelers’ behavioral intentions toward room-sharing platforms. *International Journal of Contemporary Hospitality Management*.

[35] Bucher E., Fieseler C., Lutz C. (2016). What’s mine is yours (for a nominal fee) - e. *Computers in Human Behavior*.

[36] Ahani A., Nilashi M., Ibrahim O., Sanzogni L., Weaven S. (2019). Market segmentation and travel choice prediction in Spa hotels through TripAdvisor’s online reviews. *International Journal of Hospitality Management*.

[37] Wei F., Feng N., Yang S., Zhao Q. (2020). A conceptual framework of two-stage partner selection in platform-based innovation ecosystems for servitization. *Journal of Cleaner Production*.

[38] Jiang B., Tian L. (2018). Collaborative consumption: strategic and economic implications of product sharing. *Management Science*.

[39] Shi Z., Cai R., Zhu X. (2017). Study of business model innovation for intelligent production sharing. *China Soft Science*.

[40] Lu K., Zhou J., Ju P., Xu Y. (2016). Research on pricing strategy of mobile travel platform based on bilateral market theory. *Price Theory and Practice*.

[41] ter Huurne M., Ronteltap A., Corten R., Buskens V. (2017). Antecedents of trust in the sharing economy: a systematic review. *Journal of Consumer Behaviour*.

[42] Mikalef P., Boura M., Lekakos G., Krogstie J. (2020). The role of information governance in big data analytics driven innovation. *Information & Management*.

[43] Wu X., Qiu J. (2019). A study of Airbnb listing price determinants: based on data from 36 cities in China. *Tourism Tribune*.

[44] Wang C., Chen H. (2018). Study on the price of shared short-term rental platform and its influencing factors: based on the data from xiaozhu.com. *Price: Theory and Practice*.

[45] Perez-Sanchez V., Serrano-Estrada L., Marti P., Mora-Garcia R.-T. (2018). The what, where, and why of Airbnb price determinants. *Sustainability*.

[46] Li Y., Wang S., Ma Y., Pan Q., Cambria E. (2020). Popularity prediction on vacation rental websites. *Neurocomputing*.

[47] Lawani A., Reed M. R., Mark T., Zheng Y. (2019). Reviews and price on online platforms: evidence from sentiment analysis of Airbnb reviews in Boston. *Regional Science and Urban Economics*.

[48] Wang D., Nicolau J. L. (2017). Price determinants of sharing economy based accommodation rental: a study of listings from 33 cities on Airbnb.com. *International Journal of Hospitality Management*.

[49] Ert E., Fleischer A., Magen N. (2016). Trust and reputation in the sharing economy: the role of personal photos in Airbnb. *Tourism Management*.

[50] Xu Q., Wu C., Chen Q. (2021). Dynamic optimal allocation strategy of idle resources in supply chain under shared platform. *Operations Research and Management Science*.

[51] Lunardi G. L., Maçada A. C. G., Becker J. L., Van Grembergen W. (2017). Antecedents of IT governance effectiveness: an empirical examination in Brazilian firms. *Journal of Information Systems*.

[52] Alreemy Z., Chang V., Walters R., Wills G. (2016). Critical success factors (CSFs) for information technology governance (ITG). *International Journal of Information Management*.

[53] Piscicelli L., Cooper T., Fisher T. (2015). The role of values in collaborative consumption: insights from a product-service system for lending and borrowing in the UK. *Journal of Cleaner Production*.

